# Physical abrasion method using submerged spike balls to remove algal biofilm from photobioreactors

**DOI:** 10.1186/s13104-017-2995-9

**Published:** 2017-12-02

**Authors:** Azra Nawar, Asif Hussain Khoja, Naveed Akbar, Abeera Ayaz Ansari, Muneeb Qayyum, Ehsan Ali

**Affiliations:** 1 US-PAK Centre for Advance Studies in Energy (CAS-EN), National University of Sciences and Technology (NUST) H-12, Islamabad, 44000 Pakistan; 2 Chemical Reaction Engineering Group (CREG), Faculty of Chemical & Energy Engineering, Universiti Teknologi Malaysia (UTM), Skudai, 81310 Johor Bahru, Malaysia; 3 Department of Civil and Environmental Engineering, College of Engineering, University of Massachusetts (UMass), Amherst, MA 01002 USA; 4 Punjab Bio Energy Institute, University of Agriculture, Faisalabad, 38000 Pakistan

**Keywords:** *Chlorella vulgaris*, Photobioreactor, Spike balls, Biofilm removal

## Abstract

**Objective:**

A major factor in practical application of photobioreactors (PBR) is the adhesion of algal cells onto their inner walls. Optimized algal growth requires an adequate sunlight for the photosynthesis and cell growth. Limitation in light exposure adversely affects the algal biomass yield. The removal of the biofilm from PBR is a challenging and expansive task. This study was designed to develop an inexpensive technique to prevent adhesion of algal biofilm on tubular PBR to ensure high efficiency of light utilization. Rubber balls with surface projections were introduced into the reactor, to remove the adherent biofilm by physical abrasion technique.

**Results:**

The floatation of spike balls created a turbulent flow, thereby inhibiting further biofilm formation. The parameters such as, specific growth rate and doubling time of the algae before introducing the balls were 0.451 day^−1^ and 1.5 days respectively. Visible biofilm impeding light transmission was formed by 15–20 days. The removal of the biofilm commenced immediately after the introduction of the spike balls with visibly reduced deposits in 3 days. This was also validated by enhance cell count (6.95 × 106 cells mL^−1^) in the medium. The employment of spike balls in PBR is an environmental friendly and economical method for the removal of biofilm.

## Introduction

Microalgae can be cultivated in either freshwater or saline by two different methods i.e. open or closed systems [1, 2]. Biofilm formation in PBRs is a complex process, in which algae and bacteria produce extracellular polymeric substance (EPS) to bind the cells to external surfaces of the bioreactors. *Chlorella* species are one of the most abundant producers of EPS [3]. Biofilm formation in PBRs diminishes the passage of light to algal cells which inhibits photosynthesis, hence reducing the cell growth and biomass yield.

Cleaning of the biofilm from PBRs is a challenging process, requiring a lot of effort and time. For instance, by using a cylindrical piece of foam, 10 cm long with 10% larger diameter than the inner diameter of PVC pipe, the biofilm was removed with the help of air pump that pushed the foam along the pipe required high pressure [4]. Seaweeds extract has also been used to avoid biofilm formation while cultivating green algae [5]. Antifouling booster biocides are also in practice to remove marine sediments [6]. Other available techniques include the use of ozone, ultrasonic technology, and the use of large slugs of air to intermittently scour the internal surface of the tube. Continuous circulation of close fitting balls, highly turbulent flow and suspended sand or grit particles technique have also been employed to abrade any biomass adhering to the internal surface of the bioreactor [7]. All of these processes have a set of drawbacks such as the risk of reactor tube breakage, utilization of valuable chemicals, high energy input, as well as high operating and maintenance cost, which motivates to identify feasible options for algal biofilm removal.

Spike rubber ball method is an inexpensive and novel approach for effective PBR cleaning. The spike balls have a simple design and usage, which requires no special training or extra labor costs for handling purposes. Spike balls are reusable and can be used in multiple batch processes, thereby contributing towards minimum waste generation. This technique also encourage the effective mixing of algal cultures, without posing any adverse impact on their growth cycle. This mixing effect prevents algal biomass to settle on the walls of PBR pipes, which results in the potential biofilm reduction. Another added advantage of the process is the cost-effectiveness and application in different PBRs for biofilm removal due to the availability of the spike balls in various sizes and designs.

This study is focused on devising an innovative method of removing algal biofilm from PBRs. The set of spike balls with bristles were prepared and were circulated along with the algal culture in the airlift tubular PBR as a mobile scrubber to remove biofilm leading to less deposition and high biomass production.

## Main text

### Experimental

The algal strain used in this study is *Chlorella vulgaris* (ATCC 9765), which was obtained from AlgaeTech International, Malaysia. *Chlorella vulgaris* is a photosynthetic microorganism with size ranging from 2 to 10 µm [8]. *Chlorella vulgaris* was grown in Bold’s Basal media [9] at 25 °C in a 100 mL flask which was subsequently diluted to 1000 mL with a pH of 6.5. The culture was then transferred into a 480 L PBR using electric pump for aeration whereas day light was used as a light source. The pH was monitored and adjusted to 6.5 using 2 M NaOH or 2 M H_2_SO_4_ solutions. All the chemicals used were of analytical grade from Sigma Aldrich.

**Fig. 1:**
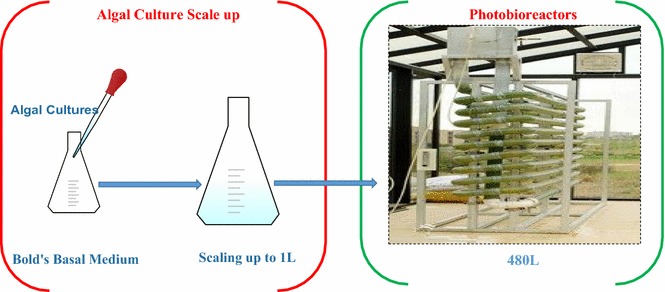
Experimental setup of vertical 480 L PBR

The vertical PBR (480 L) were used for microalgae cultivation as presented in Fig. 1. The PBR consisted of vertical and horizontal pipes, a degasser, sampling point and a pump. Submerged spike balls used in the experiment were 6 in. in diameter with 0.7 cm long projections (completely filled with water having magnets inside). The spike balls used mechanical scrubbing action against the reactors to remove deposited algal biofilms. Furthermore, the action of spike balls kept the water agitated which discouraged the algal deposition.

A small portion of reactor pipe was removed from the PBR for analysis of the biofilm characteristics. The algal biofilm present on the fragments of PBR glass pipe was air dried and later analysed using Nanovea Optical Profilometer Ps-50 (Nanovea Corp. USA) with Nanovea 3D software. *Chlorella vulgaris* cell count was monitored using Neubauer Hemocytometer (China). The cell concentration was measured using Eq. (1). Where, X_c_ is cell concentration, T_cell_ is total cells and D_f_ is dilution factor.

1 $${\text{X}}_{\text{c}} \;({\text{mL}}^{ - 1} ) = \left[ {{\text{T}}_{\text{cell}}\, (5{\text{ squares}}) \times 5000 \times {\text{D}}_{\text{f}} } \right]$$

The dry biomass (g L^−1^) was used to determine the growth rate of algal culture. The specific growth rate (r) per day was calculated using the Eq. (2) [10]. Where N_t_ and N_0_ are the dry biomass weights (g L^−1^) at time T_t_ and T_0_, respectively, and ∆t is the time interval from T_t_ to T_0_.

2 $${\text{r}} = \left[ {\frac{{{\text{Ln }}({\text{N}}_{\text{t}} ) - \ln \, ({\text{N}}_{0} )}}{{\Delta {\text{t}}}}} \right]$$

Algal yield was determined from the growth rate and doubling time. Cell doubling time (t_d_,) was estimated using Eq. (3).

3 $${\text{t}}_{\text{d}} = \left[ {\frac{\ln 2}{{{\text{r}}_{\text{max} } }}} \right]$$

### Results

**Fig. 2:**
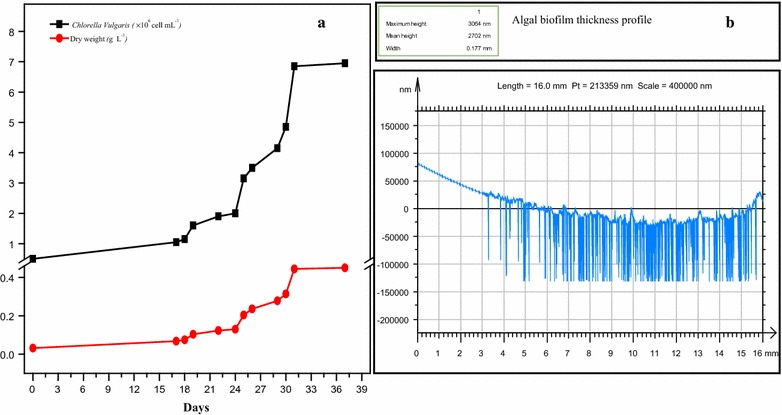
**a** Cell count and dry weight of algae. **b** Analysis of algal biofilm thickness

The algal cultivation in PBR were conducted to investigate the impact of the spike balls on the removal of produced algal biofilm. From inoculation till day 24, the cell count was observed to increase from 1.05 × 10^6^ cells mL^−1^ to 2 × 10^6^ cells mL^−1^. This number doubled in 1 day, reaching 4 × 10^6^ cells mL^−1^ on day 25, and finally peaking at 4.85 × 10^6^ cells mL^−1^ on day 30 as shown in Fig. 2a. On day 17, visible algal biofilm was seen appearing in the inner walls of PBR which kept accumulating till day 37 under the controlled growth environment. In the second PBR run, spike balls were introduced in the PBR on day 30, which resulted in an abrupt increase in cell count to 6.85 × 10^6^ cells mL^−1^. The continuation of this rise in cell count was seen until day 37. This enhancement in cell count was not only due to the result of algal growth but also dislodging of cells previously adherent to the PBR walls as more light was able to penetrate inside the PBR pipes.

Figure 2a shows the dry biomass of algae. The maximum specific growth rate (r_max_) per day was determined from all the different values of specific growth rate (r), while the maximum biomass obtained was designated as N_max_ (g L^−1^). The maximum specific growth rate (r_max_) was obtained to be 0.451 day^−1^ while the doubling time was found to be 1.5 days. As the spike balls were introduced into the PBR on day 30, a sudden increase in the cell count was observed as shown in Fig. 2a. The result obtained from Nanovea 3D software shows the thickness of algal biofilm is 3064 nm (3.064 µm) as depicted in Fig. 2b.

**Fig. 3:**
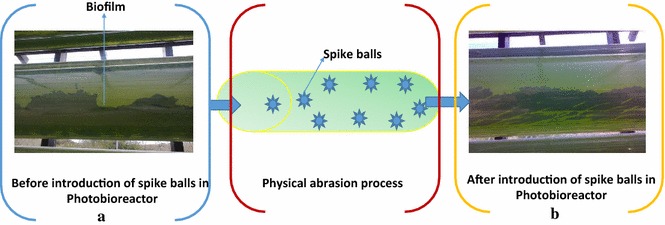
Effect of Spike balls on biofilm removal. **a** Before removal of biofilm. **b** After removal of biofilm

The spike balls were introduced into the PBR on day 30 and their effect on biofilm removal was observed by doing the visual inspection a thick layer of algal biofilm can be seen in the Fig. 3a. Nevertheless, a visible reduction in the biofilm layer was seen in 3 days after introducing the spike balls demonstrated in Fig. 3b. The image clearly show the effect of the spike balls employment into the PBR. There was a substantial effect on the amount of adherent biofilm by the brushing action of these spikes. Continues motion of these balls within the tubing ensured adequate area was covered including bottom and bends. They were easily removed via the degasser chamber and any material trapped in the projections could be cleaned with simple soap and water.

### Discussion

This study attempted the removal of biofilm on the inner walls of the PBR via physical abrasion method. Optimal growth parameters for *C. vulgaris* has already been identified and it was grown for biomass production purposes. During algal growth in PBR, the major problem faced was biofilm formation. After experimentation it was found that the algal biofilm thickness was to be 3.064 µm which is in good compromise with previously reported results [11, 12]. The spike balls removed algal biofilms significantly from the bioreactor and carried them into circulation. This increased the algal mass per liter of solution, and inhibit cells to stick in PBR, resulting biofilm formed is also nominal. The spikes on balls provide increase in surface area when in contact with walls of PBR. The scrubbing action of spikes also increases its mechanical efficiency.

While comparing with previously available technologies, few methods required high pressure to clean PBR which leads to breakage of reactor [13]. The using of ozone and ultrasound is expensive technology as it requires high energy input and it also destroys the algal cell [14, 15]. The chemical methods are also harmful for algal cell growth and expansive [16]. Whereas, the provided method is not harmful to the algal cells reactor and maintenance of the reactor. The spike ball design and preparation is very simple and no extra cost is required. The method does not required any surplus energy or chemicals, so it can be considered as environmental friendly and low cost mechanical approach to remove the biofilm form PBRs.

### Limitations

- Spike balls can get trapped in bends of PBR.
- A single spike ball design can limited to the particular reactor.
- Retrieval of damaged spike balls may be difficult.

## Abbreviations

PBR: Photobioreactor
EPS: Extracellular polymeric substance
PVC: Polyvinyl chloride
